# Extreme variation in patterns of tandem repeats in mitochondrial control region of yellow-browed tits (*Sylviparus modestus*, Paridae)

**DOI:** 10.1038/srep13227

**Published:** 2015-08-19

**Authors:** Xiaoyang Wang, Nian Liu, Hongli Zhang, Xiao-Jun Yang, Yuan Huang, Fumin Lei

**Affiliations:** 1 Co-Innovation Center for Qinba regions’ sustainable development, College of Life Sciences, Shaanxi Normal University, No. 199, South Chang’an Road, Xi’an 710062, China; 2 Key Laboratory of the Zoological Systematics and Evolution, Institute of Zoology, Chinese Academy of Sciences, 1 Beichen West Road, Chaoyang District, Beijing 100101, China; 3 College of Life Science, Datong University, Xingyun Street, Datong 037009, China; 4 State Key Laboratory of Genetic Resources and Evolution, Kunming Institute of Zoology, Chinese Academy of Sciences, No. 32, Jiaochang East Road, Kunming 650223, China

## Abstract

To investigate the evolutionary pattern and origins of tandem repeats in the mitochondrial control region of the yellow-browed tit (*Sylviparus modestus*), the control region and another four mitochondrial loci from fifteen individuals were analyzed. A 117-bp tandem repeat unit that repeated once, twice or three times in different individuals was found, and a rarely reported arrangement for this tandem repeats region that a 5′ imperfect copy at its downstream and a 3′ imperfect copy at its upstream was observed. The haplotype network, phylogenetic trees, and ancestral state reconstruction of the combined dataset of five loci suggested multiple origins of the same repeat number. The turnover model via slipped-strand mispairing was introduced to interpret the results, because mispairing occurred so frequently that multiple origins of certain repeat number were observed. Insertion via recombination should be a better explanation for the origin of this tandem repeat unit, considering characteristics of the combined sequence of the 3′ and 5′ imperfect copy, including identification of its homolog in other passerines and its predicted secondary structure.

The control region is the main non-coding region of the animal mitochondrial genome, and its length variation can affect the size of the mtDNA molecule^1^. The length of the control region is highly variable due to the appearance or absence of the variable numbers of tandem repeats (VNTRs). Tandem repeats have been widely reported in the mitochondrial control region in animals^1^, and the variation in the number of repeat units can be found among species^2^,^3^, populations^4^,^5^ or even within an individual^2^,^6^,^7^. The size and number of a tandem repeat unit differs greatly among different animals^1^. Size of repeat units can range mainly from four^8^ to approximately 200 base pairs^2^,^7^,^9^ or even more than 400 base pairs in some insects^10^,^11^,^12^, and the repeat number can range from two^7^,^12^ to more than one hundred^13^. The location of tandem repeats in control regions are mainly reported in the hypervariable domains in vertebrates^3^,^4^,^5^,^7^.

In birds, the tandem repeats have been found in several groups, predominantly at the very end of the 3′ end of the control region^8^,^14^,^15^,^16^,^17^,^18^,^19^, and are represented as simple or short sequence repeats or microsatellite-like sequences. However, only a few tandem repeats with long motifs have been found in Domain I^20^ and downstream of the Conserved Sequence Block I (CSB I box) in Domain III^3^,^4^,^20^. At these two loci, length of the repeat unit has been observed longer, commonly approximately 80 bp^3^,^20^ or sometimes even up to 128 bp^4^.

To interpret the origin and evolution of tandem repeats, mechanisms such as slipped-strand mispairing^21^, unequal crossing over^13^ or recombination^22^, and illegitimate elongation induced by the stem-loop structures in tRNAs or some non-replication origin sequences^23^ have been proposed. Considering the likelihood and frequency of occurrence, slipped-strand mispairing is considered the most likely explanation for the variable repeat numbers in mtDNA. According to this mechanism, the nascent and parental H strand competitively binds to the parental L strand during replication. When a motif in the nascent H strand misaligned to its complementary strand due to the bending or complex folding of its upstream sequence^3^,^9^,^24^, a repeated motif is formed in the following replication event. This mechanism can explain both the increase or decrease of repeat numbers and the variations in tandem repeat clusters where multiple types of repeat unit exist.

In our previous study on the complete mitochondrial genome of the yellow-browed tits (*Sylviparus modestus*)^25^, we found a 117 bp tandem repeat which repeated twice in the Domain III of its mitochondrial control region. To determine whether the number of this tandem repeat region varies among or within different individuals of this species, we sequenced the control region of 14 more individuals. We then discussed the potential evolutionary dynamics and the possible origin of this tandem repeat region by mapping the number of repeat unit in different individuals onto the phylogenetic relationships based on a combined datasets containing control region sequence and 4 more mitochondrial loci.

## Result

### Phylogenetic relationships of yellow-browed tits

**Figure 1:**
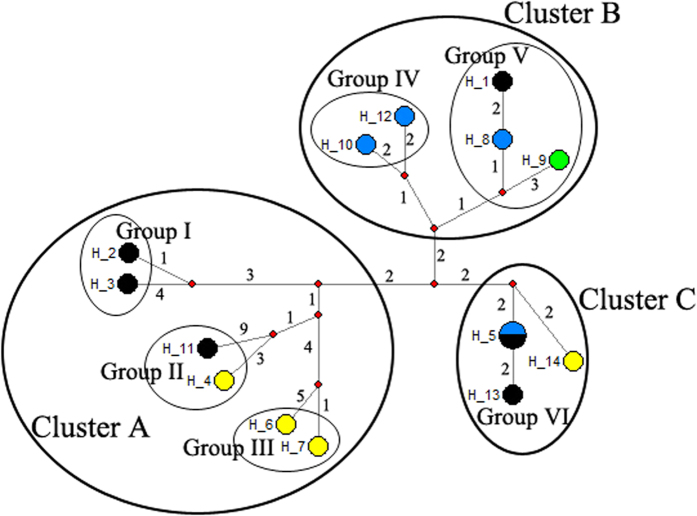
The haplotype network for the combined datasets. Colors stand for different repeat numbers: black for one, yellow (arrangement type 2A) and blue (arrangement type A + B) for two, and green for three. Numbers on the line indicate for the mutations between different haplotypes or nodes.

Five loci were amplified from all 15 surveyed individuals, and the combined dataset was 5323 bp in length (1143 bp for cyt b, 1041 bp for ND2, 684 bp for atp6, 1213 bp for COI, and 1242 bp for control region), while 55 sites were variable and 20 sites were parsimony informative. These polymorphic sites defined 14 haplotypes in all surveyed individuals, and only one haplotype was shared by two individuals (Table 1). The haplotype network of the combined dataset showed three main haplotype clusters (Cluster A, B, and C) (Fig. 1), while some haplotypes showed loosely connection, so these clusters appear to be formed by several haplotype groups. Cluster A consisted of three groups (labeled Group I-III), Cluster B was composed of two groups (labeled Group IV and V), while Cluster C contained only one group (labeled Group VI) (Fig. 1).

**Figure 2:**
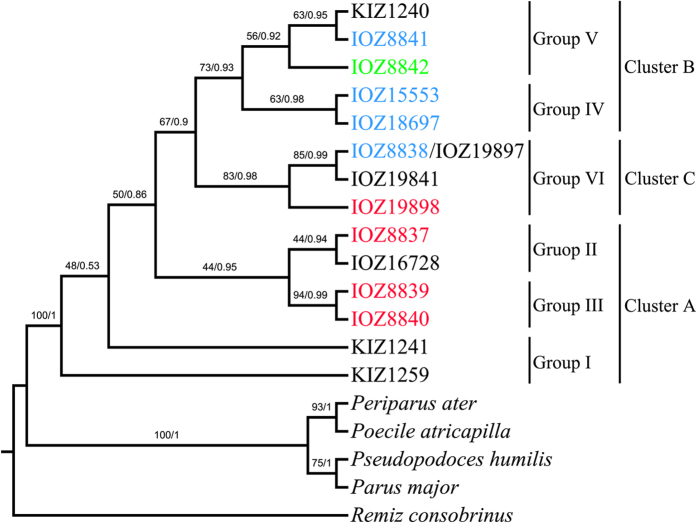
The phylogenetic tree for the combined dataset. The node supports are shown as bootstrap values from Maximum Parsimony analysis (former) and posterior probabilities for Bayesian inference analysis (latter). Colors for each individual stand for different repeat numbers of *S. modestus*: black for one, red for arrangement type 2A, blue for arrangement type A + B, and green for three.

Both Bayesian inference and Maximum Parsimony trees produced the similar tree topology, and revealed that Cluster B and Cluster C were clustered together, while Cluster A was a paraphyletic group at the basal clades (Fig. 2). For most nodes, the posterior probabilities were high, whereas the parsimony bootstrap values were slightly lower. Both Cluster A and B could be divided into stable haplotype groups as the haplotype network. Cluster B was a monophyletic cluster consisting of Group IV and V, and Group II and III of Cluster A formed a monophyletic cluster sister to Cluster B and C, while the two haplotypes in Group I were paraphyletic at the basal clades.

### Structure and content of the control region and the tandem repeat regions

**Figure 3:**
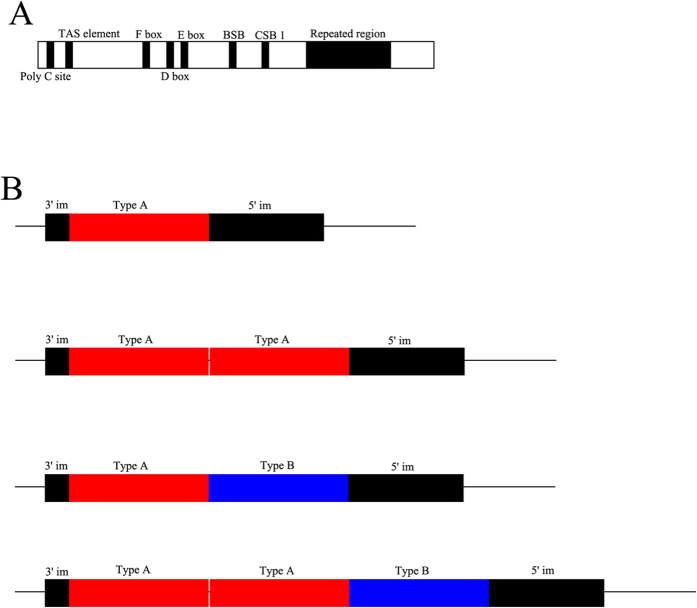
The structure for control region (**A**) and repeated region (**B**). Conserved blocks in control region marked with black and labeled at the top or bottom, and components of repeat region marked with different colors and labeled at the top.

The length of control regions of *S. modestus* varied from 1374 bp to 1609 bp, due to the number of tandem repeats and a ploy-C site at the very end of the 3′ end. The control region contained several conserved blocks: a potential termination associated sequence (TAS) element in Domain I, F box, D box, C box and BSB box in Domain II, and CSB1 in Domain III (Fig. 3A).

The tandem repeat region was found downstream of the CSB1 box in Domain III, and the numbers of tandem repeats were repeated once, twice and three times, while no heteroplasmy was found within an individual (Table 1). In the only shared haplotype, the two individuals had different numbers of repeat unit, one had only one repeat unit, while the other one had two repeat units (Table 1).

The repeat unit was 117 bp in length, and its nucleotide composition showed an unexpected high AT content (over 75%) (Table 2). At the downstream of the repeated regions, there was a 96-bp sequence that exhibited only 5 variable sites compared to the preceding 96 bp of the repeat unit (this region is hereafter termed the 5′ imperfect copy, or 5′ im copy for short, Table 2), while a 23-bp sequence at upstream of the repeated region had 6 variable sites compared to the last 23 bp of the repeat unit (hereafter termed the 3′ imperfect copy, or 3′ im copy for short, Table 2). This arrangement of the tandem repeats, with imperfect copies at both ends, has rarely been reported.

Among different individuals, two types of repeat unit with two variable sites were found (Table 2): one was found in all individuals in this study (type A), and another one was found in some individuals with two or more repeat units in which the first 22 bp at the 5′ end was identical to the 5′ im copy (type B). Four different patterns of arrangement in the repeated region were detected (Fig. 3B), three of which occurred in more than four individuals, with the exception of the pattern that repeated three times (Table 2). In all haplotype clusters and groups, all three clusters contained more than one repeat number or arrangement pattern, and three groups consisted of different repeat numbers (Fig. 1).

According to the known complete control regions from Paridae, no long repeated regions were found. However, investigation of these known parid control regions revealed a 120–121 bp motif similar to the combined 3′ and 5′ im copy (referred to the sequence showed in Table 2 hereafter) of yellow-browed tits (Table S2). The pairwise identity between the combined motif in *S. modestus* and its homologs in other parids ranged from 63.9% to 72.1%. And in some other passerine lineages, the homologs of the combined 3′ and 5′ im copy was also observed with high similarities (Table S3).

### The secondary structure of repeat regions and its adjacent sequences

Only a few short and simple stem-loop structures with low free energy (−1.1 to −3.2 kcal/M) were found in the predicted secondary structure of Type A, Type B, 3′ im copy with its upstream sequence, and 5′ im copy with its downstream sequence (Fig. S1 A–D), while no complex structures with multiple stem-loops were found in these sequences. For Type A, Type B and 5′ im copy with its downstream sequence, two stem-loop structures were formed, while for 3′ im copy with its upstream sequence, a stem-loop was formed by its 5′ end and its upstream sequence. In the regions adjacent to tandem repeats, a stem-loop structure was found ~ 20 bp upstream of the 3′ im copy, while another one was located ~ 20 bp downstream of the 5′ im copy (Fig. S1 C,D). However, the combined 3′ im and 5′ im copy sometimes formed a long stem-loop structure with several internal bulges (Fig. S1 E).

### Extreme variation in patterns of tandem repeats

**Figure 4:**
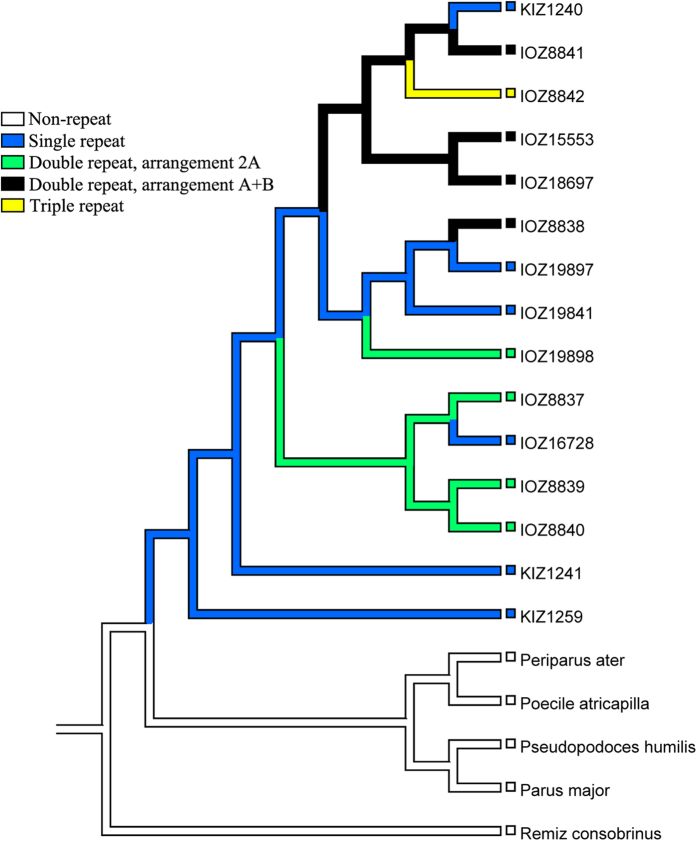
Ancestral state reconstruction of the numbers of repeat units in *S. modestus* on the phylogenetic tree under parsimony criterion. Squares stand for the observed repeat number for each individual, and lines depict the possible state between each nodes. Different colors are shown for different repeat numbers and arrangements: white for non-repeat, black for repeat once, green (arrangement type 2A) and blue (arrangement type A + B) for twice, yellow for repeat three times.

To examine whether the four arrangements of repeat region in the control region of all surveyed individuals had a single origin, the arrangement states were mapped onto the phylogenetic tree using the parsimony method. Character-mapping analysis based on the parsimony method indicated that most of the arrangement patterns had multiple origins among all surveyed individuals (Fig. 4), revealed extreme variation in the patterns of tandem repeats in the mitochondrial control region of yellow-browed tits. The possible state for each node was shown in Table S4. This result suggests that reversions among different patterns may occur frequently and that a arrangement pattern may have multiple origins.

## Discussion

Tandem duplication is one of the three possible mechanisms for the duplication of certain sections^26^, and most duplication events in mtDNAs can be explained by slipped-strand mispairing^16^,^17^,^19^,^21^. This scenario would be a better explanation why the three repeat arrangements containing two or three repeat units in yellow-browed tits. To explain how did the misalign of the nascent strand occur, some previous studies have suggested that slipped-strand mispairing might occur via the formation of stable and complex secondary structure within or among repeat units for both long and short repeat unit^3^,^9^,^24^. And in several species with long repeat units, the number of repeat units can exceed twenty^3^,^9^. In contrast, the tandem repeat unit in our study can only form two simple stem-loop structures and no inter-units structure (Fig. S1 A–C), with the repeat unit repeating three or fewer. This phenomenon may indicate that the number of repeat units could be affected by whether the complex secondary structures were formed by repeat units, and stable and complex structures would facilitate the occurrence of slipped-strand mispairing and more repeat numbers. For the tandem repeats in yellow-browed tits, the low occurrence of slipped-strand mispairing, providing few candidates for new repeat unit types, could explain the low numbers of repeat unit types and arrangements observed.

When discussing at the individual or population level, previous studies assumed that numbers of tandem repeat unit could be constant in certain phylogenetic lineages or geographical populations^4^,^5^,^6^. However, we found two conflicting results from the phylogenetic and ancestral state reconstruction results, i) different repeat numbers were observed in close related affinities, whereas the same number was shared by individuals from different phylogenetic lineages, suggesting that the same repeat number may have multiple origins; and ii) the number of repeat units were different between the two individuals from the only shared haplotype. These contradictory results among phylogenetic lineages and the repeated numbers in each surveyed individual indicated that i) the same repeat numbers in different individuals may have multiple origins as suggested by Broughton and Dowling (1997)^27^, with the same repeat number from different lineages experiencing a homogenization process; ii) although both nucleotide sequence and numbers of tandem repeats are accumulated and fixed by mtDNA replication, their evolution appear to be two inconsistent, independent processes; and iii) individuals in the only shared haplotype had different repeat numbers, suggesting that the accumulation of repeat units may be faster than that of nucleotide mutations. As the slipped-strand mispairing can occur in every replication, the change of repeated numbers can be fixed and inherited rapidly and readily, yielding different repeat numbers within a phylogenetic lineage or even within an individual (heteroplasmy).

Slipped-strand mispairing may explain the origin of most tandem repeats^20^,^21^, but it can not readily explain the origin of the tandem repeat region in the mitochondrial control region of *S. modestus*, because it can not overcome several shortcomings described below. Though the origin of both 5′ and 3′ im copy can be explained by slipped-strand mispairing^28^, this process can not readily account for the variable sites between the observed repeat unit (Type A) and the combined 3′ and 5′ im copy in yellow-browed tits, particularly the most variable 3′ im copy. Considering the alignment and the high similarities between it and its homologs in other passerines (Tables S2,S3), the combined 3′ and 5′ im copy should reflect the original sequence before its duplication. If the ancestral repeat unit was that combined sequence, the observed repeat unit (Type A in Table 2), which would have arisen after the duplication of the ancestral repeat unit and been embedded within the two ancestral repeat units, should have about 10 variable sites (comprised almost 10% of the observed repeat unit) compared with that combined sequence (the ancestral repeat unit) before next duplication. If so, as described above, some intermediate repeat units between the observed repeat unit and the combined 3′ and 5′ im copy should exist, but none were found. More importantly, the combined 3′ and 5′ im copy, together with its adjacent sequences at both ends, sometimes formed a long stem-loop structure (Fig. S1 E), causing the combined sequence to be fully embedded in the structure. This structure could also possibly prevent the duplication of the observed repeat unit via slipped-strand mispairing and resulting in a longer repeat unit than the observed repeat unit if misparing could occur via this structure.

Compared to slipped-strand mispairing, the recombination is possibly a better explanation for the tandem repeats in yellow-browed tits. Though recombination has little effect on mitochondrial genetic variability in birds^29^, both experimental and circumstantial evidence suggest it occurs pervasively in animal mtDNA^30^,^31^,^32^,^33^,^34^.

Recombination can underlie the origin of the repeated regions in yellow-browed tits, and have two possible processes. One is direct inter-molecular insertion induced by the mini-circles^33^ excised from one mtDNA molecule, and the second is a series of processes involving illegitimate elongation via the stem-loop structures in some tRNAs or some non-replication origin^23^, formation of mini circles and sequence insertions (or strand break and rejoin) via mini circles. The two processes described above require the components possessing both homologous and non-homologous recombination in mitochondria, and these components have been reported in mitochondria in several previous studies^30^,^35^,^36^. Therefore, the recombination processes occurring in mtDNA have a higher probability of occurrence.

The former one is a simple process and has been reported in some long fragment insertions^34^,^37^,^38^, whereas some studies have shown that a region adjacent to the control region is more preferentially involved in recombination induced by double-strand breaks recombination^39^. The inter-molecular recombination process via mini-circles can explain the origin of this tandem repeat sequence. And rarity of inter-molecular recombination^39^ could account for why very few tandem repeats with origins interpretable by recombination were found.

The latter one is a three-step process which can also explain the origin of the tandem repeat in yellow-browed tits clearly, but direct evidence for the whole process is lacking. This process may occur as follows. First, the short fragments induced by illegitimate elongation could be dropped from the parental strand and remain free in mitochondrial matrix. Then, the free strand exposed to the reactive oxygen for a long time, causing it to exhibit a high mutation rate^18^ or even break down into shorter fragments, and these short fragments could be cyclized as single- or double-strand mini-circles^31^ (replication may be occurred via certain stem-loop structure in these short fragments^40^,^41^). The mini circles can stably exist in the mitochondrial matrix^31^,^42^,^43^. Once the mini-circle achieved the same length and similar sequence as the observed repeat unit was cracked and linearized, the newly formed linear fragment can be inserted into the combined 3′ and 5′ im copy via recombination^31^,^34^,^37^,^38^. Thus the observed repeated unit was formed.

The yellow-browed tit was first discovered species with long tandem repeats in control regions within tits. This species was a relict, basal, and early divergent lineage in Paridae^40^, and consist of three subspecies that are discontinuously distributed in mid- or high-mountain areas of the Himalayas, southwestern China and northern Indo-China^40^. We only sampled the *S. m. modestus* in southwestern China, but the preliminary results provided starting points for a future study focusing on the variation pattern of the tandem repeats of yellow-browed tits. More samples from *S. m. modestus*, the rest two subspecies and other tits could possibly test and confirm the proposed hypothesis, and more clearly make the evolution scenario and the estimated origin time of the tandem repeat of this species and other tits.

## Method

### Sample collection, DNA extraction, PCR amplification, sequencing, and sequence annotation

Tissue samples from 15 individuals of yellow-browed tits were collected from six locations in the southwestern mountain areas of China (Table 1), and were preserved in pure ethanol and stored at −80 °C before use. The relevant specimens were kept in Institute of Zoology and Kunming Institute of Zoology and their usage were in accordance with guidelines of above institutes. All experimental protocols used in this study were approved by the Experimental Animal Ethics Committee of Institute of Zoology. Total DNA was isolated from the tissue samples using the phenol-chloroform extraction method.

To investigate the phylogenetic relationships and numbers of tandem repeat unit among *S. modestus*, we selected five mitochondrial loci, i.e., cyt b, ND2, atp6, COI, and the control region. The primers used to amplify these loci were obtained from Sorensen (2003)^45^ and Li *et al.* (2008)^46^ with some modifications (Table S1). PCRs were performed under the cycling condition as follow: 35 cycles of 93 °C for 30s, 56 °C for 30s, and 72 °C for 2min (for coding genes) or 60 °C for 4 min (for control region). The PCR products were purified and sequenced directly, and some internal primers were designed to overlap some possible gaps between poorly sequenced sections. The complete sequences were assembled and uploaded to GenBank.

Sequences were annotated through comparison to the complete mitochondrial genome of the yellow-browed tit^25^, and the locations of each gene or region were determined. The conserved blocks in avian control regions were determined following previous studies^17^,^47^. The tandem repeats in the control region were identified using Tandem Repeat Finder v4.07b.

### Sequence analyses

The sequences of the five loci (the repeated region was removed from control region) from all the 15 individuals of yellow-browed tits were aligned individually using Clustal X1.83, and the five loci were combined together by SequenceMatrix 1.7.8. The median joining networks of the combined dataset was produced by Network 4.612.

To reconstruct the phylogenetic relationships of yellow browed tits, sequences of the five loci from the 15 sampled individuals and another five tits (*Parus major* (KP137624), *Periparus ater* (KM588075), *Pseudopodoces humilis* (KP001174), *Poecile atricapilla* (KJ909190), and *Remiz consobrinus* (KC463856)) were aligned individually and combined together by SequenceMatrix 1.7.8. The phylogenetic relationships among all the surveyed individuals were rebuilt by the Maximum Parsimony method and Bayesian Inference methods implemented in PAUP*4.0 and MrBayes3.2 using the combined dataset, and the best models for Bayesian Inference method were selected by MrModeltest2.2. The parsimony method was applied to search for the shortest tree for the combined dataset using 1000 random heuristic bootstrap replicates with tree-bisection–reconnection (TBR) branch-swapping. The Bayesian inference analysis was conducted by two runs of 1 million generations, and sampled every 100 generations with a burn-in of the first 35% generations. The secondary structure of the repeat unit and its adjacent regions were predicted by MFOLD.

### Ancestral state reconstruction

Using the repeat numbers and the arrangement patterns of repeat region determined by Tandem Repeat Finder, the presence of non-repeat (0) or different repeat arrangements (1, 2, 3, 4 present for the four arrangements, repeat unit repeated once, arrangement 2A, arrangement A + B, and repeat unit repeated three times, respectively) was coded into a matrix and mapped onto the phylogenetic trees deduced from different inference methods. Ancestral state reconstructions were implemented in Mesquite 3.01 under the parsimony criterion.

## Additional Information

Accession codes: Sequences have been deposited in NCBI GenBank under Accession Numbers KT327932-KT328001. 

**How to cite this article**: Wang, X. *et al.* Extreme variation in patterns of tandem repeats in mitochondrial control region of yellow-browed tits (*Sylviparus modestus*, Paridae). *Sci. Rep.* **5**, 13227; doi: 10.1038/srep13227 (2015).

## Supplementary Material

Supplementary Information

## Figures and Tables

**Table 1:** Detailed information about each individual in this study.

**location**	**Voucher-specimen**	**Clade**	**repeated numbers**	**haplotype number for combined dataset**	**arrangement pattern**
Tengchong	KIZglgs1240	B	1	1	A
	KIZglgs1241	A	1	2	A
	KIZglgs1259	A	1	3	A
Luding	IOZ8837	A	2	4	2A
	IOZ8838	C	2	5	A + B
	IOZ8839	A	2	6	2A
	IOZ8840	A	2	7	2A
	IOZ8841	B	2	8	A + B
	IOZ8842	B	3	9	2A + B
Suiyang	IOZ15553	B	2	10	A + B
Pingwu	IOZ16728	B	1	11	A
Dujiangyan	IOZ18697	B	2	12	A + B
	IOZ19841	C	1	13	A
	IOZ19897	C	1	5	A
	IOZ19898	C	2	14	2A

Note: The haplotype number of each individual is identical with the network in Figure 1; IOZ, Institute of Zoology, Chinese Academy of Sciences; KIZ, Kunming Institute of Zoology, Chinese Academy of Sciences.

**Table 2:**
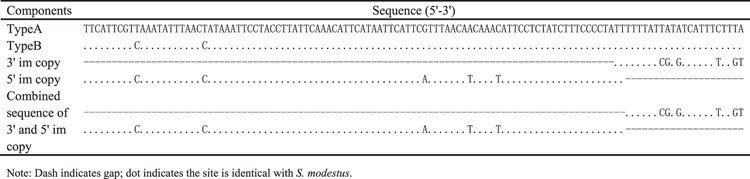
Alignment of the two repeat units, 3′ and 5′ im copy of *Sylviparus modestus*.
