# Supplementary material for: Extreme variation in patterns of tandem repeats in mitochondrial control region of yellow-browed tits (Sylviparus modestus, Paridae)
Source: Sci Rep. 2015 Aug 19;5:13227. doi: 10.1038/srep13227 (PMC4541255; doi:10.1038/srep13227)
Supplement: Supplementary Information [file srep13227-s1.doc]

Extreme variation in patterns of tandem repeats in mitochondrial control region of yellow-browed tits (*Sylviparus modestus*, Paridae)

Xiaoyang Wang^1^, Nian Liu^1^, Hongli Zhang^3^, Xiaojun Yang^4^, Yuan Huang^1,*^, Fumin Lei^1,2,*^

Supplementary Information

Figure S1 Secondary structure deduced from Mfold for A) Type A; B) Type B; C) 5' im copy and its adjacent sequence; D) 3' im copy and its adjacent sequence; E) combined 3' and 5' im copy.


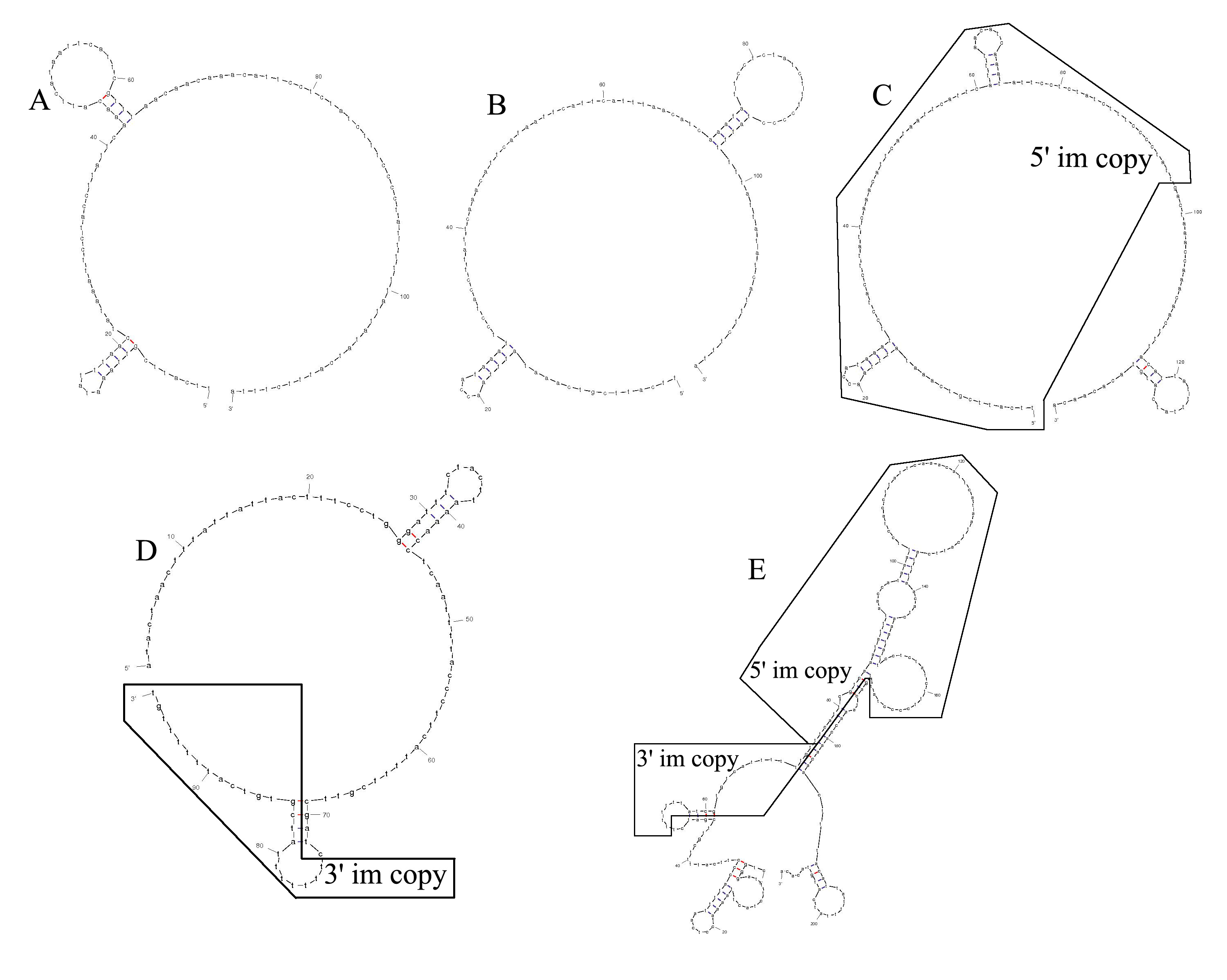


Figure S2 Numbers for each node on the phylogenetic tree used in ancestral state reconstruction.


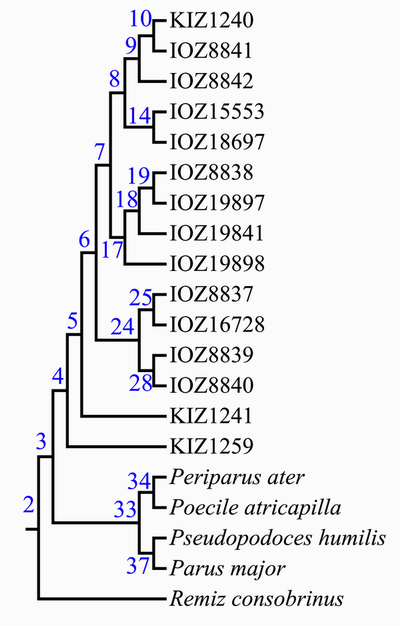


Table S1 The PCR primers used in this study.

| Target | Primer | Sequence | Locus | Reference |
| --- | --- | --- | --- | --- |
| cyt b | L14770 | TAGGCCCAGAAGGACTAGC | ND5 | Sorenson et al. 1999 |
|  | H16064 | CTTCAATCTTTGGCTTACAAGACC | tRNA^Thr^ | Sorenson et al. 1999 |
| ND2 | L5143 | GAACCTGCACCAAAGGGATCAAAAC | tRNA^Gln^ | Sorenson et al. 1999 |
|  | H6313 | ACTCTTGTTTAAGGCTTTGAAGGC | tRNA^Trp^ | Sorenson et al. 1999 |
| COI | L6615 | CCTCTGTAAAAAGGACTACAGCC | tRNA^Tyr^ | Sorenson et al. 1999 |
|  | H7956 | GGGTAGTCCGAGTAACGTCG | COI | Sorenson et al. 1999 |
| atp6 | L9230 | ACCACAACCACTACCCCTTG | atp8 | Li et al. 2008 |
|  | H10163 | AAATCATGCCGTAGCGTAGG | COIII | Li et al. 2008 |
| control region | L16225 | CCGAGACAACCCACGCACAAG | ND6 | Sorenson et al. 1999 |
|  | H614 | GGRAARATGCCGCGATYACG | control region | Sorenson et al. 1999 |
|  | L16525 | ACAAACACCACCAACATCCCCCC | ND6 | Sorenson et al. 1999 |
|  | H1530 | GTGGCTGGCACAGGATTTACC | 12S rRNA | Sorenson et al. 1999 |

Note: The underline nucleotides were modified from Sorenson (2003) and Li et al. (2008); degenerated base pairs followed the IUPAC codes: R=A/G, Y=C/T.

Table S2 Alignment of the combined 3' and 5' im copy of *Sylviparus modestus* and its homologs in other parids.

| Species | Sequence (5'-3') |
| --- | --- |
| *S. modestus* | TTTTATCGTGTCATTTTTTGTTTCA----TTCGTCAAATATTTAACC-ATAAATTCCTACCTTATTCAAACATTCATAATTCATTCATTTAACATCAAATATTCCTCTATCTTTCCCCTATT |
| *P. montanus* U85657 | .CA.G..A.T.TTA.....TAA...AAAT...A.T.........G.T-C..T.CG.....A.C.AC...G..C....C.....C-.T.CGC...C....C.......AGA...........A |
| *C. caeruleus ogliastrae* AF068742 | G.G.CATT.T.TT......T.A...AAAT...A.....C.....G.TGT....CG...G..CCG.C.....G..T..C......-..A..C...C....C........GG......A.CCAA |
| *C. c.caeruleus* AF068741 | G.G.CATT.T.TT......T.A...AAAT...A.....C.....G.TAT....CG......CCG.C.....G..T..C......-..A..C...C....C........GG......A.CCAA |
| *P. major* AF059662 | ....CATT.T.AT......T.A...AACT...A..........G..T-.....CG......CC.AC........T..C..C...-T.A..T........C..................C.CC |
| *P.cinctus* AF257185 | .CG.G..A.T.TTA.....TAA.T.AAAT.....T.........G.T-C..T.CG.....A...AC...G..CA...C......-...CAC...C....CG........A........C..A |
| *P. atricapilla* KJ909190 | NNNNNNNNNNNNNNNNNNNNAA...AAAT..TA.T.........GGT-C..T..G.....A.C.AC.......C...C..C...-.....C...CT...C..C....C.A........C..A |
| *Periparus ater* KM588075 | .CA.G..A.T.TTA.....TAA...AAAT...A.T.......C.G.T-C....CG.....A...A.......CC...C...T..-.....C.T.CAC..C........GCT.......CGCA |

Note: Underlined nucleotides of *S. modestus* stand for the 3' im copy, while the rest nucleotides of *S. modestus* stand for the 5' im copy; dash indicates gap; dot indicates the site is identical with *S. modestus*.

Table S3 Alignment of the combined 3' and 5' im copy of *Sylviparus modestus* and its homologs in other passerines lineages.

| Species | Sequence (5'-3') |
| --- | --- |
| *S. modestus* | TTTTATCGTGTC-ATTTTTTGTTTCATTCGTCAAA-TATTTAACCATAAATTCCTACCTTATTCAAA-CATTCATAATTCATTCATTTAACATCAAATATTCCTCTATCTTTCCC-CTATT |
| *Pinicola enucleator* KM078781 | .......T..A.-...........TT..-..T...AA...A.......TC.C.....A..T.C....-..........CG..C.....T.A.CT..-C........GCT...T.TAG.... |
| *Erithacus akahige* AB720096 | .......T..A.-...........T.AA.A.....A.T........A.TT.......AAAG-A....C....T..C..CAC.CA..CA..A.CA..-...........T......-.C... |
| *Loxops caeruleirostri* KM078776 | .......T..A.-...........TT..T..T...AA...A......TT..C.....A..T.A....CT......C..CA...T....T.A..T..-CT........AG.....TA..... |
| *Pseudonestor xanthoph* KM078809 | .......T..A.-...........TT..-..T...AA...A......TT..C.....A..T.A....CT......C..CA...T....T.AG....-CC........AA.....TA....A |
| *Hemignathus parvus* KM078799 | .......T..A.T...........TT..T......AA...A......TT..C.....A..T.A....T.......C..CA...T....T.A..T..-CT.......GAG.....TA....C |
| *Fringilla coelebs* AF002972 | .......T..A.-...........TT..TA.....AA.ACA.......TTC......A..T.C....A.......C.........CCATCA..T..-CT.........T...T.TAA..AA |
| *Cyanolyca armillata* FJ598192 | ......TT...TAT...AACAAAAT.AA.AA....-CT..GT.TA..TTTAG....GA....C....-....A..C....G........CAT....-.T............C...CTA.C. |
| *Carduelis pinus* KM078796 | .......T..A.-...........TT.C-..T...AA...A......TT..C.....A..T......T..A.......CA..C...A.T.A..T..-CC.......GCA...T.TAA..AC |
| *Carpodacus erythrinus* KM078766 | .......T..A.-...........TT..T..T...AA...A......TT........A..T.C....T.......C..CA..CA..A.TGA..T..-C........G.A.....TG....A |

Note: Underlined nucleotides of *S. modestus* stand for the 3' im copy, while the rest nucleotides of *S. modestus* stand for the 5' im copy; dash indicates gap; dot indicates the site is identical with *S. modestus*.

Table S4 The possibilities for each arrangement state on each node. The most possible states were bolded. Nodes numbers were shown in Figure S2.

|  | 10 | 9 | 14 | 8 | 19 | 18 | 17 | 7 | 25 | 28 | 24 | 6 | 5 | 4 | 34 | 37 | 33 | 3 | 2 |
| --- | --- | --- | --- | --- | --- | --- | --- | --- | --- | --- | --- | --- | --- | --- | --- | --- | --- | --- | --- |
| Non repeat | 0.025 | 0.030 | 0.007 | 0.031 | 0.015 | 0.016 | 0.025 | 0.032 | 0.015 | 0.005 | 0.017 | 0.033 | 0.043 | 0.122 | **0.993** | **0.993** | **0.967** | **0.785** | **0.850** |
| Arrangement A | 0.341 | 0.207 | 0.021 | 0.214 | **0.757** | **0.803** | **0.437** | **0.425** | 0.323 | 0.025 | 0.279 | **0.523** | **0.852** | **0.813** | 0.003 | 0.003 | 0.018 | 0.154 | 0.077 |
| Arrangement 2A | 0.032 | 0.055 | 0.013 | 0.108 | 0.035 | 0.084 | 0.415 | 0.314 | **0.632** | **0.960** | **0.664** | 0.345 | 0.068 | 0.032 | 0.002 | 0.002 | 0.005 | 0.022 | 0.025 |
| Arrangement A+B | **0.499** | **0.405** | **0.945** | **0.527** | 0.177 | 0.080 | 0.091 | 0.176 | 0.016 | 0.005 | 0.024 | 0.068 | 0.021 | 0.018 | 0.002 | 0.002 | 0.005 | 0.019 | 0.024 |
| Arrangement 2A+B | 0.103 | 0.303 | 0.014 | 0.120 | 0.016 | 0.017 | 0.032 | 0.053 | 0.014 | 0.005 | 0.017 | 0.031 | 0.015 | 0.016 | 0.002 | 0.002 | 0.005 | 0.019 | 0.024 |
